# A 5-Year-Old Female With an Aneurysmal Bone Cyst of the Proximal Humerus

**DOI:** 10.7759/cureus.23761

**Published:** 2022-04-02

**Authors:** Ashwin Sivakumar, Ozo Akah, Lakshmi Sai Chintala, Abimbola O Ajibowo, Aadil Khan

**Affiliations:** 1 Medicine, Angeles University Foundation (AUF), Angeles, PHL; 2 Internal Medicine, Carnegie Mellon University (CMU), Houston, USA; 3 Internal Medicine, Vydehi Institute of Medical Sciences and Research Centre, Bangalore, IND; 4 Internal Medicine, Lugansk State Medical University, Luhansk, UKR; 5 Surgery, Lala Lajpat Rai (LLR) Hospital, Kanpur, IND

**Keywords:** lytic bone lesion, curettage, skeletal tumor, proximal humerus, aneurysm bone cyst

## Abstract

An aneurysmal bone cyst (ABC) is a non-malignant, skeletal tumor that is extremely rare and most commonly presents within the first two decades of life. Genetic mutation of the *USP6* gene on chromosome 17 remains to be the most commonly accepted reasoning as ABC’s etiology remains unknown. As the radiographic appearance of ABC is quite similar to other kinds of bone cysts, a histological diagnosis is often required to attain a definitive diagnosis. Curettage remains the gold standard treatment with high chances of local recurrence. Evidence has shown the beneficial applications of administering a sclerosing agent. Further trials would improve the level of evidence available for physicians to make a better management plan. We have demonstrated the treatment of an aneurysm bone cyst of the proximal humerus on a 5-year-old female in this report, which might be utilized as a reference for future procedures.

## Introduction

An aneurysmal bone cyst (ABC) is a non-malignant, blood-filled, rare skeletal tumor first described by Jaffe and Lichtenstein in 1942 due to its cyst-like appearance inside the bone. They are most commonly presented as an expanding mass and are often painful. Children below the age of 20 have an increased predisposition to develop ABC due to their rapid growth rate. Since the cause of ABC is known, the most widely accepted reasoning is the genetic mutation of the ubiquitin-specific peptidase 6 (*USP6*) gene on chromosome 17. Although ABCs are the primary cause of presentation in 70% of cases and secondary associations of other tumors in 30% of cases, the primary presentation has a low incidence rate of 0.14 to 0.32 per 100,000 individuals [1]. 

Plain radiography can be used to diagnose ABC at an initial stage. A radiolucent cystic lesion inside the metaphyseal region of the bone is the characteristic radiographic appearance of ABC. A biopsy of the cyst with a histological confirmation showing hemorrhagic tissue with fibrous septa containing spindle cells, inflammatory cells, some amount of giant cells, and with or without a rim of osteoblasts is needed to proceed to management. The lesion is damaging and has the potential to spread into the surrounding cortical bone if left untreated. Curettage and bone grafting of the affected area remains the gold standard for managing ABC while the use of other experimental methods like sclerotherapy, radionuclide ablation, and en bloc resection have shown some evidence in management [2].

In this report, we have shown the management of an ABC of the proximal humerus of a 5-year-old female, which can be used as a source of reference for future practices.

## Case presentation

A five-year-old female patient presented with pain and swelling over the right shoulder for two months following an injury to the right shoulder. There was no other significant contributing history. The pain was acute in onset, non-radiating, aggravated on movement, and relieved by rest and medication. The swelling was insidious in onset, progressive, tender, defined, not associated with a scar or draining sinus, and not adherent to the underlying skin. The movement was painful and restricted over the right shoulder.

**Figure 1:**
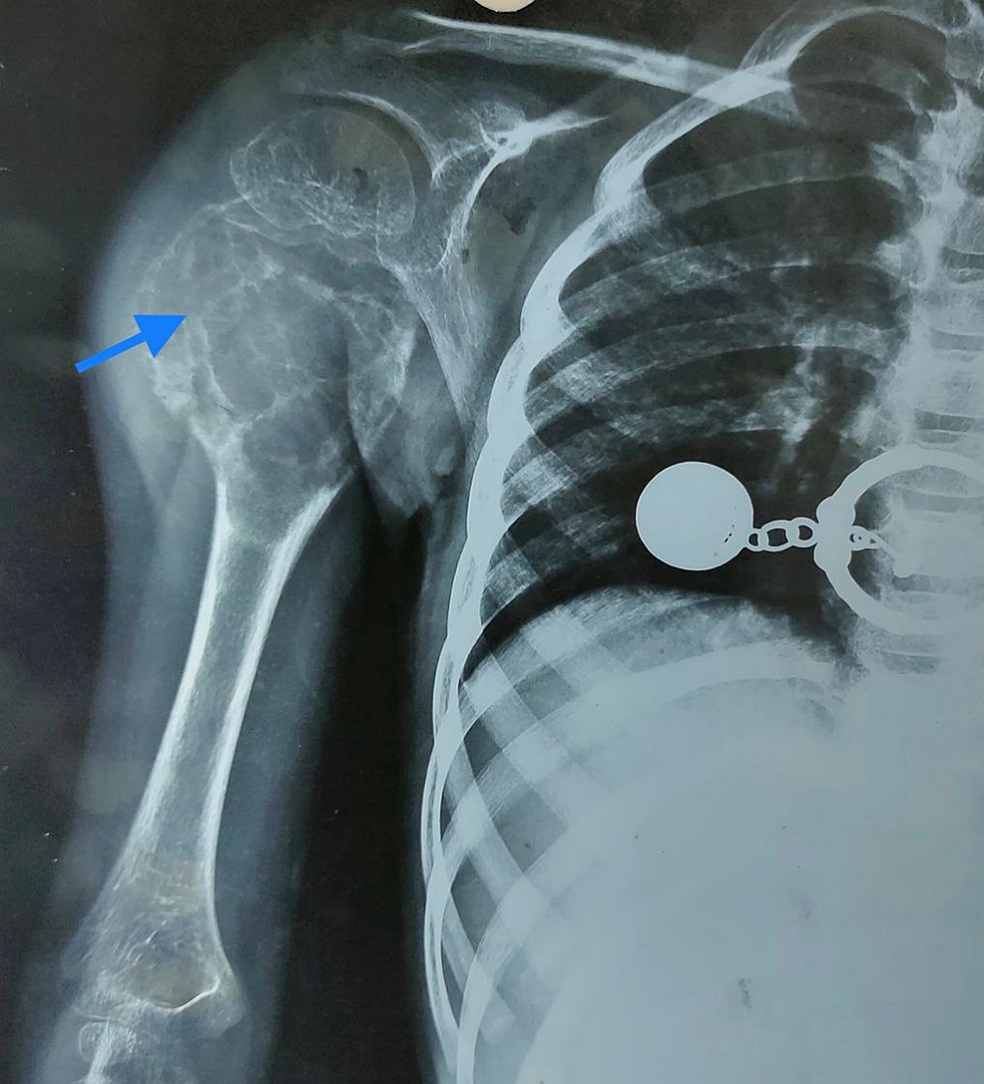
Plain radiograph (anteroposterior view) showimg a lytic lesion in the proximal portion of the right humerus.

The plain radiograph revealed a well-defined, expansile, lytic lesion involving the proximal shaft of the humerus region approximately 12 cm x 10 cm in size ( Figure 1).

**Figure 2:**
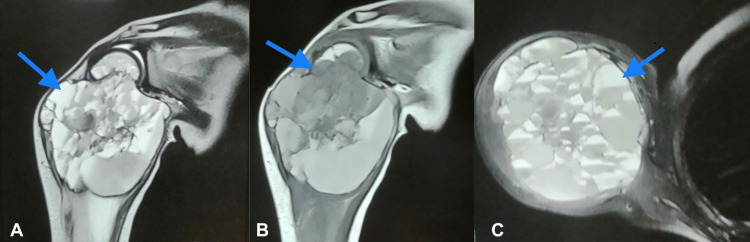
MRI T1/T2-weighted of right shoulder showing cystic lesion arising from proximal shaft humerus in coronal section (A, B) and axial section (C).

An MRI of the right shoulder reveals a well-defined, oval-shaped, altered signal intensity cystic lesion arising from the proximal shaft of the humerus. The lesion shows the mixed hypointense and hyperintense areas on both t1/t2w images with multiple hypointense internal septations. The overlying cortex is intact with no periosteal thickening or reaction. The lesion measured approximately 6.9 (cc) x 5.1 (tr) x 5.9 (ap) cm (Figure 2) in dimensions; subsequently, we ordered a fine needle aspiration cytology (FNAC) of the right shoulder, which showed a benign vascular lesion. All findings were suggestive of an aneurysmal bone cyst, owing to which we did septolysis and debulking of the lesion. Polidocanol, a sclerosing agent, was injected percutaneously. We treated conservatively and followed up.

**Figure 3:**
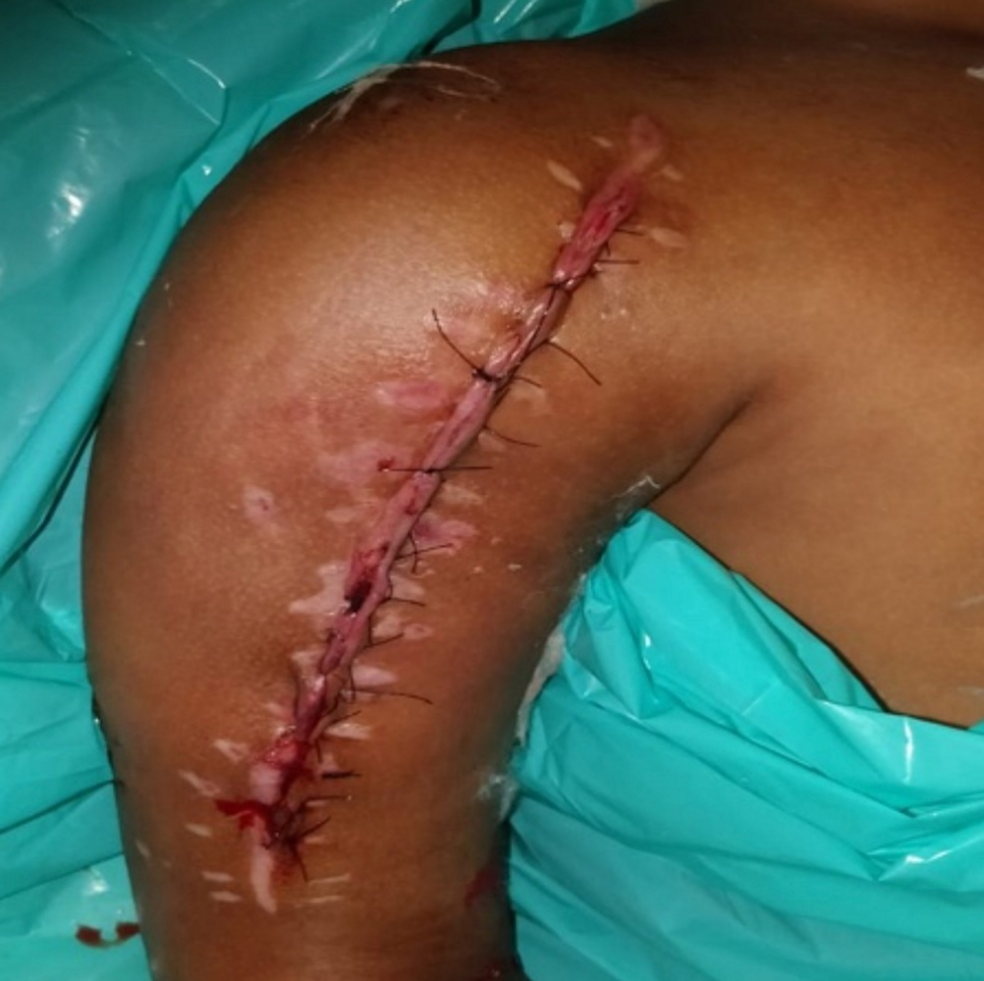
Postoperative view after excision and grafting.

**Figure 4:**
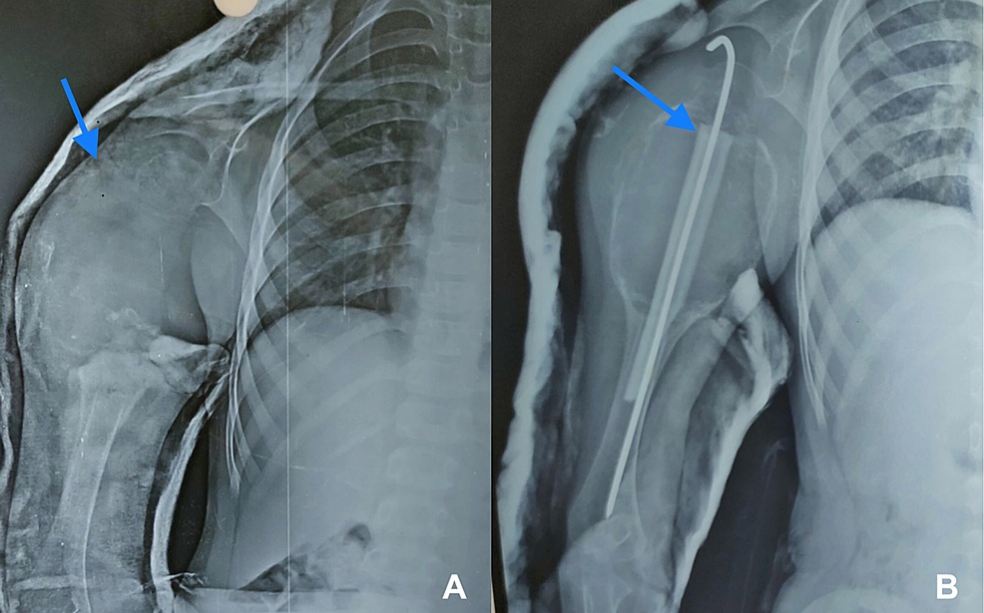
Preoperative (A) and postoperative (B) radiograph (anteroposterior view) showing curettage of the lesion with bone grafting and fixation with K-wire. K-wire: Kirschner wire

At follow-up after five months, she presented with a more aggressive swelling. Plain radiograph and MRI were ordered, which showed a large well-defined, expansile, multiseptate, proximal humeral mass with multiple fluid-level. Written informed consent was taken and the patient was prepared for surgery. Extensive tumor excision and curettage were done (Figure 3) and the bone defect was filled with an autogenous cancellous fibular bone graft, along with prophylactic fixation with Kirschner wires (K-wire) (Figure 4). A bone biopsy was sent for histopathological examination, which showed an aneurysmal bone cyst.

**Figure 5:**
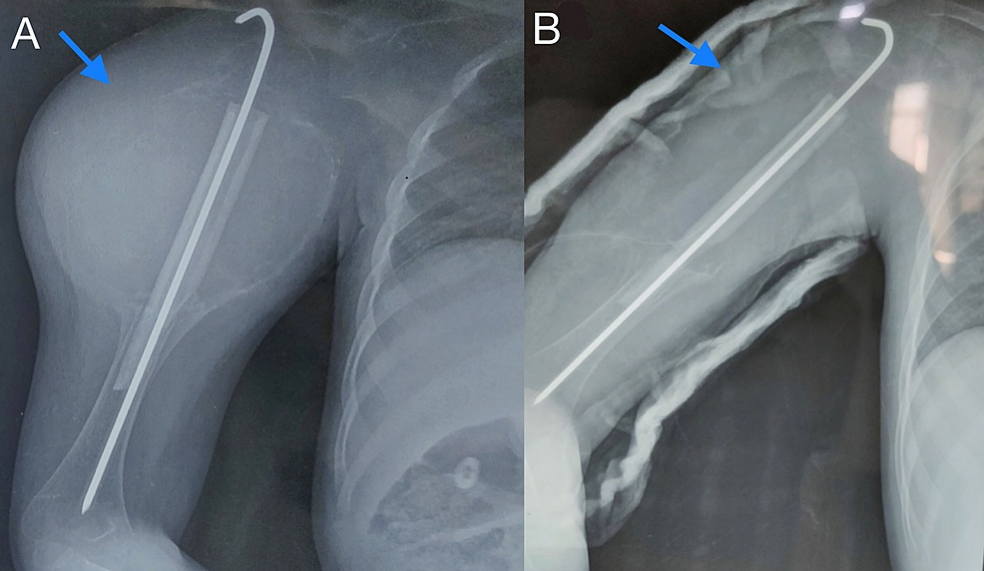
Preoperative (A) and postoperative (B) plain radiograph at 10-month follow-up showing excision and curettage of the tumor followed by chemical cauterization.

After 10 months, the patient again had the same complaints. Radiographs were repeated, which showed a recurrence of the lesion. Again, informed consent for surgery was obtained followed by excision and curettage of the lesion, and chemical cauterization was done with phenol (Figure 5). She was kept on U-Slab for two months and active movement of fingers was advised. After 12 weeks, x-rays showed healing of the lesion and incorporation of bone graft, after which the patient was allowed full weight-bearing on her right arm, and range of motion on the right shoulder was fully painless and unrestricted.

**Figure 6:**
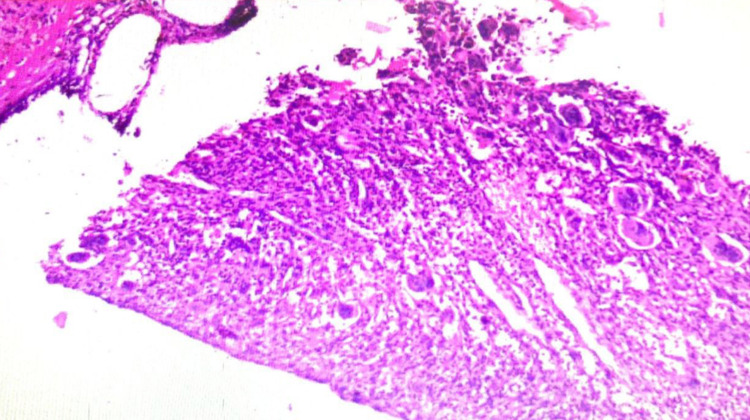
Magnification (40x) images of H&E-stained tissue histology slides showing stroma consisting of proliferative fibroblasts, spindle cells, areas of osteoid formation, and uneven large cystic spaces. H&E: hematoxylin and eosin

The histopathological examination of the specimen showed large spaces filled with blood separated by cellular septae containing fibrovascular tissue, a large number of osteoclastic giant cells, and inflammatory cells. In the septae, there are foci of osteoid tissue seen. At the place, there is degenerating calcified fibromyxoid tissue also seen. All findings were suggestive of the giant cell-containing lesion with a strong possibility of an aneurysmal bone cyst (Figure 6).

## Discussion

The accurate reason for the development of an ABC is debatable but ABCs are known to undergo different developmental stages as part of their natural maturation process. It starts with a lesion that damages certain areas of bone called an osteolytic lesion. Later, it matures into a mature cyst, which in due course may attain a late or calcified stage. The progression of ABCs is unpredictable as the growth level ranges from aggressive to slow, but most of them attain a mature state and they rarely regress [3]. Even though some amount of evidence suggests that having low signal T1 images and high-intensity T2 images are highly suggestive for the diagnosis of ABC instead of a simple bone cyst (SBC) [4], the diagnosis should always be confirmed histologically. Diagnosing an aneurysm bone cyst with radiological evidence has yielded inaccurate results, especially in differentiating among similar cysts. In a study involving 18 radiologically-based diagnoses of SBC, only 12 cases were histologically confirmed to be SBC.; the other six were found to be ABC [5]. 

In spite of being a rare disease, the management of ABC has relatively robust literature information. The gold standard for treating ABC is curettage. Administering sclerosing agents has shown promising results in terms of cure rate, tumor healing (Enneking classification), and functional score. A study done with 72 patients has shown a cure rate of 97%. Studies involving pediatric cases for sclerotherapy have also shown similar results [6,7]. One of the most dreaded complications of ABC is recurrence. A study reviewing 65 patients showed that local recurrence after curettage is more than 50% and adjuvant phenol is not associated with local recurrence [8]. Contradicting the previous evidence, our patient showed lesion healing on x-rays after the administration of adjuvant phenol. An alternative method for simple curettage by combining a high-speed burr and electrocautery has shown a reduced local recurrence rate but was dismissed due to its weak evidence and sample size [9]. Experimental treatment options like *USP6*-targeted therapy and *RANKL*-targeted therapy are still in developmental stages and need further testing to prove their clinical use and efficacy.

## Conclusions

An ABC is a highly destructive benign bone tumor with an unfavorable prognosis if not treated early, despite surgical treatment. Proper follow-up should be scheduled at least every three months for the first two years of treatment. In our patient, we surgically treated the tumor first by sclerotherapy after which we curetted and filled it with autogenous bone graft followed by chemical cauterization in order to restrict the aggressive tumor from causing a pathological fracture. The patient responded well to the treatment and is under follow-up.
